# Efficient, Decentralized Detection of Qualitative Spatial Events in a Dynamic Scalar Field

**DOI:** 10.3390/s150921350

**Published:** 2015-08-28

**Authors:** Myeong-Hun Jeong, Matt Duckham

**Affiliations:** 1 CyberGIS Center for Advanced Digital and Spatial Studies, University of Illinois at Urbana-Champaign, Urbana, IL 61801, USA; 2 School of Mathematical and Geospatial Sciences, RMIT University, Melbourne, Victoria 3000, Australia

**Keywords:** geosensor networks, decentralized spatial computing, surface networks, critical points, coordinate-free algorithm, spatial event detection, environmental monitoring

## Abstract

This paper describes an efficient, decentralized algorithm to monitor qualitative spatial events in a dynamic scalar field. The events of interest involve changes to the critical points (*i.e.*, peak, pits and passes) and edges of the surface network derived from the field. Four fundamental types of event (appearance, disappearance, movement and switch) are defined. Our algorithm is designed to rely purely on qualitative information about the neighborhoods of nodes in the sensor network and does not require information about nodes’ coordinate positions. Experimental investigations confirm that our algorithm is efficient, with O(n) overall communication complexity (where *n* is the number of nodes in the sensor network), an even load balance and low operational latency. The accuracy of event detection is comparable to established centralized algorithms for the identification of critical points of a surface network. Our algorithm is relevant to a broad range of environmental monitoring applications of sensor networks.

## 1. Introduction

Our geographic world is highly dynamic, and consequently, monitoring change over space is of considerable interest in many scientific communities. Geosensor networks have a particularly important role in environmental monitoring [1]. These wireless networks of sensor-enabled computers embedded in the geographic environment can help with capturing information about change and even responding to events. This paper is concerned specifically with qualitative spatial events connected to the critical points (peaks, pits and passes) of a monitored dynamic scalar field, such as a temperature, humidity, soil moisture or pollution field.

Critical points in a scalar field are points with zero slope: peaks, pits and passes [2]. Critical points can be connected by critical edges (e.g., ridges connecting peaks and passes; channels connecting passes and pits). The combination of critical points and edges forms a surface network, also known as a Morse–Smale complex [3,4]. These intuitive network structures capture the essential features of complex surfaces.

Applications of geosensor networks to monitoring events in dynamic scalar fields must negotiate the unique resource constraints of geosensor networks. Limited resources favor algorithms that can operate in-network without centralized control by minimizing communications. Further, energy resource constraints may prevent positioning (e.g., as might be captured using GPS) or positioning systems may be unavailable (e.g., dense vegetation area). In addition, the limited granularity of geosensor networks imposes restrictions on the capability to infer information about surface networks and, so, events occurring on a surface network.

Based on these limitations, recent research has yielded decentralized algorithms that are capable of identifying critical points and edges in a static field monitored by a geosensor network [5,6,7]. However, to date, this work has not addressed the problem of efficiently and accurately identifying events occurring on monitored surface networks. Building on this previous research, this paper: (1) provides a rigorous definition of the fundamental events that can occur on surface networks; and (2) develops and tests a decentralized and coordinate-free algorithm to capture those events efficiently.

Section 2 begins by examining in more detail the strengths and limitations of previous research on monitoring qualitative events in a dynamic field. The formal model of a geosensor network and the extended definitions of critical points are defined in Section 3, leading to the design of a decentralized algorithm for identifying events at critical points in a dynamic scalar field (Section 4). Section 5 presents an experimental evaluation of our algorithm in terms of its the efficiency and accuracy using simulation. The results confirm the efficiency and effectiveness of the approach, discussed in Section 6. The paper concludes with a summary and suggestions for future work in Section 7.

## 2. Background

Events are defined as salient changes in state. An important question in this study is then: what events can occur on a dynamic scalar field monitored by a geosensor network? In short, what changes in state are salient for surface networks?

One of the most frequently-cited works relevant to this question is [8]. The authors of [8] present a formal analysis of the evolution of Reeb graphs on $S^{3}$ for time-varying data. The approach is based on Jacobi sets [9], which delineate the paths that critical points take over time. A more empirical approach was taken by the author of [10], who identified primitive events occurring on surface networks and analyzed changes in retail activities based on these primitive events on surface networks. Both of these approaches defined primitive events as the appearance and disappearance of critical points and “switch” where the connectivity of the network edges changes. The author of [10] additionally defined as events the movement of critical points.

Although these works do identify salient changes to surface networks, in practice, none is directly applicable to computation within a geosensor network. The approach of [8] relies on Jacobi sets, based, in turn, on smooth, continuous functions. Such functions are known not to be suited to real-world data [11,12], such as the discrete observations derived from a geosensor network. The analysis of [10] relies on centralized computation and interpolation based on the geometry of the surface, which conflicts with our requirements for a decentralized and coordinate-free computing environment.

Broadening the search, an alternative to investigating events in surface networks is to look instead at events in regions and their boundaries. Surface networks partition a scalar field into regions. Each region contains those locations in the “catchment” of a unique peak and pit combination. As a scalar field evolves, these regions change and evolve. This paper focuses on the evolution of catchments to monitor events occurring on surface networks, because the catchments can be used as spatial structures to collect global information about the entire system in a geosensor network.

Analysis of the geometry (such as the volume or centroid) of regions [13,14,15] leads to identifying appearance, disappearance, merging and splitting as four primitive events that can occur in regions. The authors of [16,17] also arrive at these four primitive events, using the topology of Reeb graphs to track the evolution (sequences of events) of burning regions. Closely related, the authors of [18] use contour trees along with geometric information about the volumes of regions to monitor essentially the same four events in turbulent vortex structures. The analysis of [19,20] of primitive events, involving simple, connected polygons, yields two further event types: expansion and contraction. Although the specific terms used to name these six events vary across papers, others have similarly arrived at these six events, including a range of applications in disciplines, such as meteorology [21,22], and tracking the evolution of social groups [23,24].

In terms of surface networks, however, distinctions between the events’ splitting and appearance and, by symmetry, the events’ merging and disappearance are not meaningful. It is purely a matter of interpretation whether a new peak, say, “split” from a pre-existing peak or independently sprang into existence (“appeared”). Thus, in the context of surface networks, this previous work suggests up to four primitive events: merging/disappearance or splitting/appearance of critical points and expansion and contraction of the regions associated with those critical points.

Other approaches are also possible. Unlike [19,20], the authors of [25] also consider disconnected regions (*i.e.*, consisting of more than one simple polygon) to yield a more discerning framework consisting of nine different events, additionally defining movement, as well as distinguishing two types of merging and of splitting based on the geometric characteristics of the regions. The authors of [26] provide the pure topological events: appearance, disappearance and two types of merging and splitting, distinguishing merging and splitting involving regions with holes and without holes. However, in the case of scalar fields in the plane, it is not possible for a surface network to partition the space into regions with holes (although this is a possibility in surface networks in other embedding spaces, such as a torus).

In summary, the key events relevant to surface networks appear to be: appearance (or splitting), disappearance (or merging) and movement of critical points; and switching of the edges connecting critical points. However, none of the approaches encountered are directly applicable to our decentralized and coordinate-free computing environment. With the exception of [26], none of the approaches described above are decentralized. However, as we have seen, the authors of [26] include several events for regions that are not directly relevant to surface networks. Further, most of the approaches above rely on geometric information about the surface and, so, are ultimately reliant on access to coordinate information about the location of critical points and their associated regions.

This paper does, however, substantially revise and extend our previous work in [7]. Our previous work defines and evaluates a decentralized and coordinate-free algorithm to identify critical points and surface networks in a static field. Based on the extended definitions of discrete surface networks, this paper not only defines basic spatial events occurring on surface networks, but also provides a decentralized algorithm to detect these events in a dynamic field.

## 3. Model

Based on our review of the existing literature relevant to events on surface networks, we will now proceed with the design of an algorithm capable of detecting our four primitive surface network events: appearance, disappearance, movement and switch. The algorithm is amenable to decentralized computation and capable of operating within the constraints of the limited spatial granularity of a sensor network.

### 3.1. Algorithm Preliminaries

The formal model of a geosensor network in this paper follows the approach of [27]. A geosensor network is modeled as a undirected graph G=(V,E), where *V* is the set of sensor nodes and E⊆V×V is the set of direct, one-hop communication links between the neighboring nodes. The set of neighboring nodes is represented as a function $\mathrm{nbr}:V\to 2^{V}$, where $\mathrm{nbr}\left(v\right)=\left\{v^{\prime }|v,v^{\prime }\in E\right\}$. Each node has a unique identity, modeled as a function id:V→N. Each node also has the ability to sense its changing environment, modeled using the sense function, sense:V×T→R. For example, $\mathrm{sense}(v_{1},t_{5})=16$ °C indicates that node $v_{1}$ sensed a value of 16 °C at time $t_{5}$. Note that although we allow time-varying sensed data, we assume that the structure of the communication graph and location of the nodes are static.

Using this foundational model of a geosensor network, the algorithm definitions in subsequent sections follow the decentralized algorithms design and specification style of [27,28]. In brief, there are four key components of decentralized algorithms: restrictions, system events, actions and system states. Restrictions concern the assumptions made about the environments in which an algorithm will operate. For example, there are no restrictions in terms of the structure of the communication networks in our algorithm. We do not require spatial information, such as coordinate information. However, we assume our sensor networks are static and the communications are reliable (see Algorithm 1, Line 1). **Algorithm 1** Update gradient vectors.1: Restrictions: Geosensor network, G=(V,E); sensor function sense:V×T→R; communication neighborhood $\mathrm{nbr}:V\to 2^{V}$; identifier function id:V→N; reliable communication.2: State transition system: 〈{idle,peak,pitx},{(idle,peak)),
(idle,pitx)}〉3: Local variables: the last sensing value, $s_{l}$, initialized empty; list of upstream neighbors, Nu, initialized empty; list of downstream neighbors, Nd, initialized empty; an ascent vector, av, initialized empty; the last ascent vector, ${\mathrm{av}}_{l}$, initialized empty; a strong peak id, pkid, initialized empty; a last peak id, ${\mathrm{pkid}}_{l}$, initialized empty; a strong pit id, ptid, initialized empty; neighbors’ peak identifiers, Pkcell, initialized empty; neighbors’ pit identifiers, Ptcell, initialized empty; a weak peak’s ascent bridge, ${\mathrm{wpk}}_{av}$, initialized empty;IDLE, PEAK, PITX4: When
$\overset{˚}{\mathrm{sense}}$ changes5:  **set**
$s_{l}:=\mathrm{sense}$- -
*Save the last sensed value*6:  **broadcast** (upd8, $\overset{˚}{\mathrm{id}}$, $\overset{˚}{\mathrm{sense}}$, pkid, ptid)IDLE7: *Receiving* (upd8, *i*, *s*, $c_{pk}$, $c_{pt}$)8:  **update**
Nu and Nd- -
*Based on s value*9:  **update**
Pkcell and Ptcell10:  **if**
|Nu|>0 and |Nd|>0
**then**11:   **set**
av:=max(Nu)- -
*Likewise for descent vector*12:   **if**
pkid≠av’s peak identifier **then**13:    **set**
pkid:=av’s peak identifier14:    **broadcast** (udsf, $\overset{˚}{\mathrm{id}}$, pkid, ptid, "channel")15:  **if**
|Nu|=0
**then***- -Likewise for Nd*16:   **set**
${\mathrm{av}}_{l}:=\mathrm{av}$ and **set**
av:=∅17:   **if**
$|{\mathrm{wpk}}_{av}|>0$
**then**18:    **set**
${\mathrm{av}}_{l}:={\mathrm{wpk}}_{av}$ and **set**
${\mathrm{wpk}}_{av}:=\emptyset$19: *Receiving* (udsf, *i*, $n_{pk}$, $n_{pt}$, sf)20:  **if**
sf="channel"
**then**21:   **update**
Pkcell- -
*Update a neighbor’s peak**identifier*22:   **if**
i=av and $n_{pk}\neq \mathrm{pkid}$
**then**23:    **set**
$\mathrm{pkid}:=n_{pk}$24:    **broadcast** (udsf, $\overset{˚}{\mathrm{id}}$, pkid, ptid, "channel")

System events define the external stimuli that nodes can respond to, such as receiving a message from another node or sensing a change to a monitored environmental variable (see Algorithm 1, Line 4 or 7). When a system event occurs, a node will react by initiating an atomic, terminating sequence of operations, called an action (see Algorithm 1, Line 6). System states allow nodes to respond to the same events with different actions based on the effects of previous system events and actions (see Algorithm 1, Line 3 or 6).

Finally, in our algorithms, the over-dot notation ($\overset{˚}{\mathrm{sense}}$) is used to refer to the current node’s knowledge of the sense function. (*i.e.*, $\overset{˚}{\mathrm{sense}}$ equals sense(v) where node v∈V). This notation helps maintain a clear distinction between each individual node’s local knowledge and the global state of the network.

### 3.2. Discrete Surface Networks

This section explains the definitions of critical points for the finite spatial granularity of the discrete point data that are generated by a geosensor network. These definitions will be used in the following Section 4 for the algorithm explanation.

**Figure 1:**
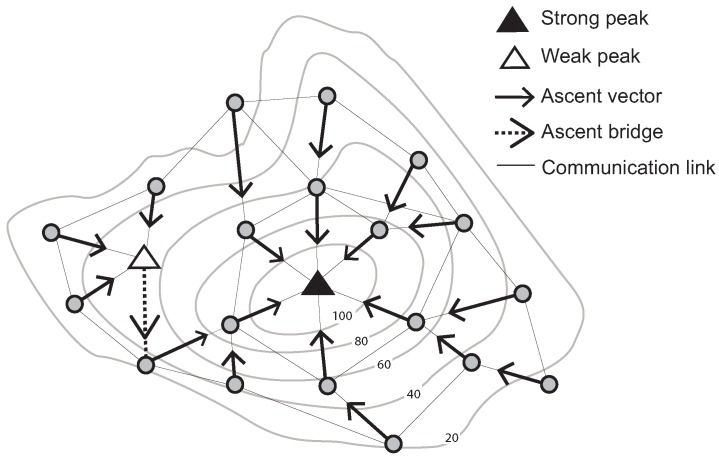
Identification of a strong and a weak peak, ascent vectors and an ascent bridge (Contour lines describe a scalar field showing the difference in elevation between consecutive contour lines. The sensed values can be estimated using a contour map).

The ascent (descent) vector of a node is defined as the unique directed edge from that node to its one-hop neighbor with the highest (lowest) sensed value of all neighbors. A “strong peak” is defined as a node *v*, such that: (a) all neighbors of *v* have a lower sensed value than *v*; and (b) the ascent vectors of all neighbors of *v* point to *v*. Conversely, a “weak peak” satisfies Condition a, but does not satisfy Condition b. For example, a weak peak in Figure 1 has communication links with four neighboring nodes. These nodes’ sensed values are lower than that of the weak peak. However, one of its neighboring nodes’ ascent vectors does not point to the weak peak.

In addition, a weak peak can be connected to a strong peak via an ascent bridge. An ascent bridge is an edge from a weak peak *v* to a neighboring node whose ascent vector points away from *v* (or symmetrically for a weak pit, strong pit and descent bridge). These basic structures are illustrated in Figure 1.

Using this information, it is then possible to design decentralized algorithms to identify for each node the (strong) peak and pit associated with that node (*i.e.*, found by following a chain of ascent/descent vectors from that node). In effect, this will partition the sensor network into regions (“catchment areas”).

**Figure 2:**
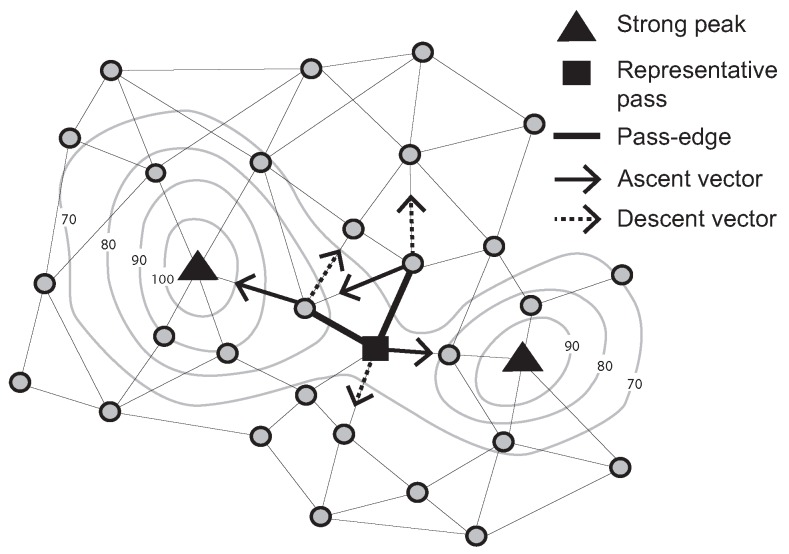
Representative pass (Contour lines describe a scalar field showing the difference in elevation between consecutive contour lines. The sensed values can be estimated using a contour map.)

Further, a “pass-edge” is defined as a pair of adjacent nodes that are each associated with different peaks and pits. Examples of pass-edges are shown in Figure 2. Two thick black lines are pass-edges for which associated peaks and pits are different (*i.e.*, two ascent and descent vectors between a pair of adjacent nodes indicate different peaks and pits).

Due to network granularity, there may be multiple candidate pass-edges for the same peak/pit pairs. Multiple pass-edges were grouped together as a pass-cluster. Moving to a dynamic scenario, however, it becomes highly inefficient to continually monitor events occurring on a group of pass-edges. Therefore, in this paper, we add a further definition of the representative pass amongst a pass-cluster. For example, let *R* be the set of pass edges that connect two specified pairs of peaks and pits in the surface network. Further, let pe_incident(v) denote the number of pass edges in *R* incident with a node *v*. A representative pass is chosen to be the unique node *r*, such that there exists no *v* with pe_incident(v)>pe_incident(r) (*i.e.*, no other node *v* is incident with a greater number of pass edges); and for any *v* where pe_incident(v)=pe_incident(r), then sense(r)>sense(v).

A representative pass is at the “center” of the multiple pass-edges, somewhat analogous to the centroid of multiple edges. In Figure 2, the filled-square symbol indicates a representative pass, because this node has two pass-edges for the the same peak/pit pairs. Focusing on a representative pass, rather than a potentially large set of pass-edges, it becomes easier and more efficient to monitor events occurring on passes.

It is possible that two representative passes occur as one-hop neighbors, akin to a monkey saddle in a continuous surface. In practice, such monkey saddles do occur in our sensor networks, but due to network granularity effects, rather than being a true reflection of the topography of the underlying surface. In other words, monkey saddles typically occur as a result of adverse network connectivity leading to certain spatially nearby nodes not being one-hop network neighbors. Thus, in our algorithms, we also include procedures for coordination amongst neighboring representative passes to account for such granularity effects. In these cases, we select a single representative pass (termed a “strong pass”) from amongst the group of neighboring representative passes, in a similar fashion to that used to identify a representative pass from amongst neighboring pass edges.

In addition to a monkey saddle, it is important to note that degenerate critical points can occur due to the same values. Such a plateau is mainly generated by the discrete quantization while extracting surface networks. The authors in [4,29] deal with degenerate critical points by using perturbation. However, real data from geosensor networks are unlikely to contain identical sensed values. This paper assumes that there are no plateaus between one-hop neighbor nodes.

## 4. Algorithm

As argued in Section 2, there are four primitive events occurring on surface networks: appearance, disappearance, movement and switch. Previous approaches to monitoring such events (e.g., [8,10]) are based on centralized computation and require geometric information. In keeping with the resource constraints imposed by sensor networks, in this paper, we develop a decentralized algorithm that can monitor surface events without coordinate information. For the ease of explanation, we present first the monitoring of events on peaks and pits and then the monitoring of events on passes.

### 4.1. Monitoring Events Occurring on Peaks and Pits

This section examines the design of a decentralized algorithm for monitoring events occurring on peaks and pits. The following subsection addresses the problem of monitoring events on passes.

The network is initialized by decentrally identifying strong peaks and pits. In brief, each node broadcasts its sensed value. Nodes can then locally determine their ascent and descent vectors and whether they are a peak or pit. Flooding of a single initialization message from each identified peak and pit can then be used to enable every node in the network to be informed of its unique (strong) pit and peak, as well as discern apart weak and strong peaks and build gradient (ascent and descent) bridges. The initialization is linear in the number of nodes in the network, requiring 3|V|+m messages, where m≪|V|.

**Figure 3:**
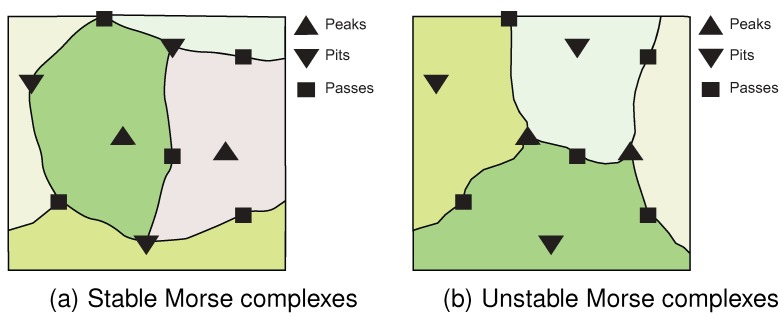
Stable/Unstable Morse complexes. This figure is adapted from [30].

The result of initialization is to partition the nodes into regions. Nodes in each region are associated with a unique pair of peak and pit identifiers. These regions are called stable/unstable Morse complexes [30] (Figure 3).

Stable/unstable Morse complexes can be regarded as catchment areas. As the dynamic field evolves, our algorithm operates by inspecting these catchment areas for changes that indicate events occurring on the surface network. For example, the appearance and disappearance of peaks and pits can be detected by monitoring the changes occurring in those critical points’ catchment areas. If one catchment area is divided into two between consecutive time steps, this indicates that a new peak has appeared on the surface network. Conversely, if two catchment areas are merged into one between consecutive time step, this indicates that one of the peaks has disappeared from the surface network.

Based on catchment areas, it is now possible to specify a decentralized spatial algorithm to monitor all of the events occurring on peaks and pits. For the ease of explanation, this part of the algorithm is split into four components (Algorithms 1–4) based on the types of events occurring. In the sequel, we also only discuss the case for peaks; identification of events for pits occurs in a symmetric fashion.

#### 4.1.1. Algorithm 1: Update Gradient Vectors

Algorithm 1 responds to changes in the dynamic field. Each node monitors locally any changes in its sensed value. When a change is detected, a node broadcasts an update message (upd8) to its neighbors (Algorithm 1, Line 6). Based on the neighbors’ sensed values, the neighbors may, in turn, update their ascent and/or descent vectors. If a node needs to update its peak identifier (*pkid*) following a change in its ascent vector, it must then initiate a cascade of notifications about this change to its neighbors (Algorithm 1, Lines 12–14). Detecting changes in gradient vectors in this way provides the basis for all higher level monitoring of the events occurring on critical points (Algorithm 1, Lines 19–24).

#### 4.1.2. Algorithm 2: Monitor Peak Movement

Using Algorithm 2, peak movement is deduced from changes to the nodes’ gradient vectors. A node that transitions from state peak (a strong peak) to state idle (a non-peak) indicates that a peak has moved. Such transitions are detected in Algorithm 2. In summary:

1. A node in a peak state that detects a new ascent vector (a higher neighbor) sends a wipk message to its ascent neighbor and transitions to an idle state (Algorithm 2, Lines 34–40).
2. A wipk message is forwarded along the ascent vector until it reaches a peak (Algorithm 2, Lines 9 and 12).
3. The peak receiving an wipk message evaluates whether it is a strong or a weak peak (Algorithm 2, Line 7). If it is a strong peak, this node transitions to a PEAK state (Algorithm 2, Line 27). If not, the node unicasts the wipk message via its ascent bridge (Algorithm 2, Line 29) and the algorithm continues from Step 2 above.

**Algorithm 2** Monitoring a peak movement.1: Fragment extend: Algorithm 12: Local variables: the flag for receiving wipk messages, bpk, initialized false; neighbors adjacent to a potential peak, Tcells, initialized empty;IDLE3: *Receiving* (wipk, $p_{s}$, *i*, $p_{pk}$)4:  **if**
$\mathrm{pkid}=p_{pk}$
**then**5:   **if**
|Nu|=0
**then**6:    **set**
bpk:= true7:    **broadcast** (etcl, $\overset{˚}{\mathrm{id}}$)- -
*To identify a strong peak*8:   **else**9:    **send** (wipk, $p_{s},\overset{˚}{\mathrm{id}},\mathrm{pkid}$) to av or ${\mathrm{wpk}}_{av}$10:  **else**11:   **if**
|Nu|>0
**then**12:    **send** (wipk, $p_{s},\overset{˚}{\mathrm{id}},\mathrm{pkid}$) to av or ${\mathrm{wpk}}_{av}$13: *Receiving* (etcl, *i*)14:  **if**
$\overset{˚}{\mathrm{av}}=i$
**then**15:   **send** (cetc, $\overset{˚}{\mathrm{id}}$, true, pkid, $\overset{˚}{\mathrm{sense}}$) to a node *i*16:  **else**17:   **send** (cetc, $\overset{˚}{\mathrm{id}}$, false, pkid, $\overset{˚}{\mathrm{sense}}$) to a node *i*18: *Receiving* (cetc, *i*, *bflag*, $n_{pk}$, $n_{s}$)19:  **if**
bflag=false
**then**20:   **set**
${\mathrm{wpk}}_{av}:=\left\{i,n_{pk},n_{s}\right\}$- -
*Set ascent bridge*21:  **if**
i∉Tcells
*Tcells*
**then**22:   :=Tcells∪{i}23:  **if**
|Tcells|=|Nd|
**then**24:   **if**
|Nu|=0
**then**25:    **if**
(|av|>0 and $|{\mathrm{wpk}}_{av}\left|=0\right)$ or (|av|=0 and $|{\mathrm{wpk}}_{av}\left|=0\right)$
**then**- -
*A strong peak*26:     **set**
av:=∅; **set**
bpk:=false; **set**
Tcells:=∅27:     **become** PEAK28:    **if**
$|{\mathrm{wpk}}_{av}|>0$
**then**- -
*A weak peak*29:     **send** (wipk, $p_{s},\overset{˚}{\mathrm{id}},\mathrm{pkid}$) to ${\mathrm{wpk}}_{av}$30:   **else**31:    **send** (wipk, $p_{s},\overset{˚}{\mathrm{id}},\mathrm{pkid}$) to av or ${\mathrm{wpk}}_{av}$PEAK32: *Receiving* (upd8, *i*, *s*, $c_{pk}$, $c_{pt}$)33:  **update**
Nu, Nd, Pkcell, and Ptcell34:  **if**
|Nu|>0
**then**35:   **set**
av:=max(Nu)36:   **if**
pkid≠av’s peak identifier **then**37:    **set**
pkid:=av’s peak identifier38:    **broadcast** (udsf, $\overset{˚}{\mathrm{id}}$, pkid, ptid, "channel")39:   **send** (wipk, $\overset{˚}{\mathrm{id}},\overset{˚}{\mathrm{id}},\mathrm{pkid}$) to av40:   **become** IDLE


#### 4.1.3. Algorithm 3: Monitor Peak Disappearance

It is similarly straightforward to detect peak disappearance (Algorithm 3). When a PEAK node transitions to an IDLE state, it forwards a wipk message along its ascent vector (see Algorithm 2). If this wipk message reaches a node that has a different peak identifier, that node can then infer that the peak represented by the node that initiated the wipk message has disappeared. Algorithm 3 can be summarized as follows:

1. When a wipk message reaches a PEAK node, the node checks if the peak identifier contained in that message matches its own peak identifier. If not, this event triggers the PEAK node to return a rwpk message back down the ascent vector to the origin of the wipk message (Algorithm 3, Line 5 or Line 24).
2. When the origin of a wipk message subsequently receives a rwpk message, it confirms a peak disappearance. The node then broadcasts swpk messages that trigger the update of peak identifiers (Algorithm 3, Line 12) in nodes below it in the surface.

**Algorithm 3** Monitoring a peak disappearance.1: Fragment extend: Algorithm 1, 2IDLE2: *Receiving* (wipk, $p_{s}$, *i*, $p_{pk}$)3:  **if**
$\mathrm{pkid}\neq p_{pk}$
**then**4:   **if**
|Nu|=0
**then**5:    **send** (rwpk, $p_{s},\overset{˚}{\mathrm{id}},\mathrm{pkid},p_{pk}$) to *i*6: *Receiving* (rwpk, $p_{s}$, *i*, $p_{pk}$, $p_{pkl}$)7:  **if**
$\overset{˚}{\mathrm{id}}=p_{s}$ and $\mathrm{pkid}=p_{pk}$
**then**8:   A peak identifier has been already changed because of udsf messages.9:  **if**
$\overset{˚}{\mathrm{id}}=p_{s}$
**then**10:   **if**
$\mathrm{pkid}\neq p_{pk}$
**then**11:    **set**
$\mathrm{pkid}:=p_{pk}$- -
*Peak disappearance*12:    **broadcast** (swpk, "disappearance", $\overset{˚}{\mathrm{id}}$, pkid, $p_{pkl}$)13:   **update**
Pkcell- -
*Update neighbors’ peak**identifier*14:  **else**15:   **send** (rwpk, $p_{s},\overset{˚}{\mathrm{id}},p_{pk},p_{pkl}$) to a node sent wipk message16: *Receiving* (swpk, e, *i*, $p_{pk}$, $p_{pkl}$)17:  **if**
e="disappearance"
**then**18:   **if**
$\mathrm{pkid}=p_{pkl}$
**then**19:    **set**
$\mathrm{pkid}:=p_{pk}$- -
*Set new peak id*20:    **broadcast** (swpk, “disappearance", $\overset{˚}{\mathrm{id}}$, pkid, $p_{pkl}$)21:   **update**
PkcellPEAK22: *Receiving* (wipk, $p_{s}$, *i*, $p_{pk}$)23:  **if**
$\mathrm{pkid}\neq p_{pk}$
**then**24:   **send** (rwpk, $p_{s},\overset{˚}{\mathrm{id}},\mathrm{pkid},p_{pk}$) to *i*

Interestingly, when a node that was previously a peak receives a rwpk message, it may have already changed its peak identifier: udsf messages may change that node’s peak identifier before a rwpk message is returned to it (Algorithm 3, Line 8). In this case, this message simply confirms that a peak disappeared.

#### 4.1.4. Algorithm 4: Monitor Peak Appearance

Lastly, Algorithm 4 presents a mechanism to monitor the appearance of peaks. As is common in decentralized algorithm design, we make no assumptions in our algorithm about network synchronization (such as message ordering or bounded communication delays). Combined with the lack of centralized control inherent in decentralized algorithms, this lack of coordination makes it more challenging to monitor a peak appearance, when compared to events such as peak movement or disappearance. When monitoring peak movement or disappearance, the node that was previously a peak can assist in the event detection (*i.e.*, by sending a wipk message). However, there are no such triggers for inferring peak appearance. Each node could locally deduce whether it is a peak by comparison of its sensed value with those of its neighbors. However, the approach proves impractical, because delays to upd8 messages from neighbors lead to numerous “false alarms”. The lack of synchronization frequently leads a node incorrectly inferring that it is a peak based only on partial information about its neighbors. Furthermore, without assuming bounded communication delays, there are no guarantees as to how long each node must wait for update messages from its neighbors. Therefore, a different approach is taken to infer peak appearance in Algorithm 4.

1. If a node detects pit movement, it broadcasts wnpk messages in order to infer peak appearance. (Algorithm 4, Line 5).
2. When a node receives a wnpk message from a descent neighbor (*i.e.*, via a descent vector, *dv*), it delivers a wnpk message to its ascent neighbor (*i.e.*, via an ascent vector, *av*). By following each node’s ascent vector, this message reaches a peak (Algorithm 4, Line 10).
3. If a node receives a wnpk message from a downhill node with the same pit identifier (but not a descent vector), this node broadcasts wnpk messages to its neighbors to trigger a wnpk message to peaks. These nodes usually exist on boundaries where two catchment areas meet (Algorithm 4, Line 12).
4. If a node with the highest value among neighbors takes a wnpk message in an IDLE state, it will first check whether it is a strong peak or a weak peak (Algorithm 4, Line 20). If it is a strong peak, this node will broadcast swpk messages to create a new catchment area (*i.e.*, a new peak appearance; Algorithm 4, Line 35).
5. If a node receives a swpk message from an ascent neighbor, it will update its peak identifier that is indicated in the swpk message. It then broadcast an swpk message in order to trigger the construction of the new catchment area (Algorithm 4, Line 43).

**Algorithm 4** Monitoring a peak appearance.1: Fragment extend: Algorithm 1, 2, 32: Local variables: the flag for receiving wnpk messages, bnpk, initialized false;IDLE3: *Receiving* (cebc, *i*, *bflag*, $n_{pt}$, $n_{s}$)4:  **if** A pit movement is confirmed **then**5:   **broadcast** (wnpk, $\mathrm{ptid},\overset{˚}{\mathrm{id}},\mathrm{false}$)6: *Receiving* (wnpk, $n_{pt}$, *i*, flag)7:  **if**
|Nu|>0 and bnpk=false
**then**8:   **if**
flag=false
**then**9:    **if**
(|dv|>0 and i=dv) or $(|{\mathrm{wpt}}_{dv}|>0$ and $i={\mathrm{wpt}}_{dv})$
**then**10:     **send** (wnpk, ptid, $\overset{˚}{\mathrm{id}}$, false) to av or ${\mathrm{wpk}}_{av}$11:    **else**12:     **broadcast** (wnpk, $\mathrm{ptid},\overset{˚}{\mathrm{id}},\mathrm{true}$)13:    **set**
bnpk:=true14:   **else**15:    **if**
i∈Nd and $\mathrm{ptid}=n_{pt}$
**then**16:     **send** (wnpk, ptid, $\overset{˚}{\mathrm{id}}$, false) to av or ${\mathrm{wpk}}_{av}$17:     **set**
bnpk:=true18:  **if**
|Nu|=0 and bnpk=false and bpk=false
**then**19:   **set**
bnpk:=true; **set**
Tcells:=∅20:   **broadcast** (ispk, $\overset{˚}{\mathrm{id}}$)21: *Receiving* (ispk, *i*)22:  **if**
$\overset{˚}{\mathrm{av}}=i$
**then**23:   **send** (rspk, $\overset{˚}{\mathrm{id}}$, true, pkid, $\overset{˚}{\mathrm{sense}}$) to a node *i*24:  **else**25:   **send** (rspk, $\overset{˚}{\mathrm{id}}$, false, pkid, $\overset{˚}{\mathrm{sense}}$) to a node *i*26: *Receiving* (rspk, *i*, *bflag*, $n_{pk}$, $n_{s}$)27:  **if**
bflag=false
**then**28:   **set**
${\mathrm{wpk}}_{av}:=\left\{i,n_{pk},n_{s}\right\}$29:  **if**
i∉Tcells
**then**30:   *Tcells*
:=Tcells∪{i}31:  **if**
|Tcells|=|Nd|
**then**32:   **if**
|Nu|=0 and bpk=false
**then**33:    **if**
(|av|>0 and $|{\mathrm{wpk}}_{av}\left|=0\right)$ or (|av|=0 and $|{\mathrm{wpk}}_{av}\left|=0\right)$
**then**- -
*A strong peak*34:     **set**
av:=∅; **set**
bpk:=false; **set**
Tcells:=∅ ; **set**
$\mathrm{pkid}:=\overset{˚}{\mathrm{id}}$35:     **broadcast** (swpk, "appearance", $\overset{˚}{\mathrm{id}}$, pkid, $p_{pkl}$)36:     **become** peak- -
*Peak appearance*37:    **if**
$|{\mathrm{wpk}}_{av}|>0$
**then**- -
*A weak peak*38:     A weak peak appearance39: *Receiving* (swpk, *e*, *i*, $p_{pk}$, $p_{pkl}$)40:  **if**
e= “appearance” **then**41:   **if**
$\mathrm{pkid}=p_{pkl}$ and av=i
**then**42:    **set**
$\mathrm{pkid}:=p_{pk}$43:    **broadcast** (swpk, "appearance", $\overset{˚}{\mathrm{id}}$, pkid, $p_{pkl}$)

#### 4.1.5. Summary

**Figure 4:**
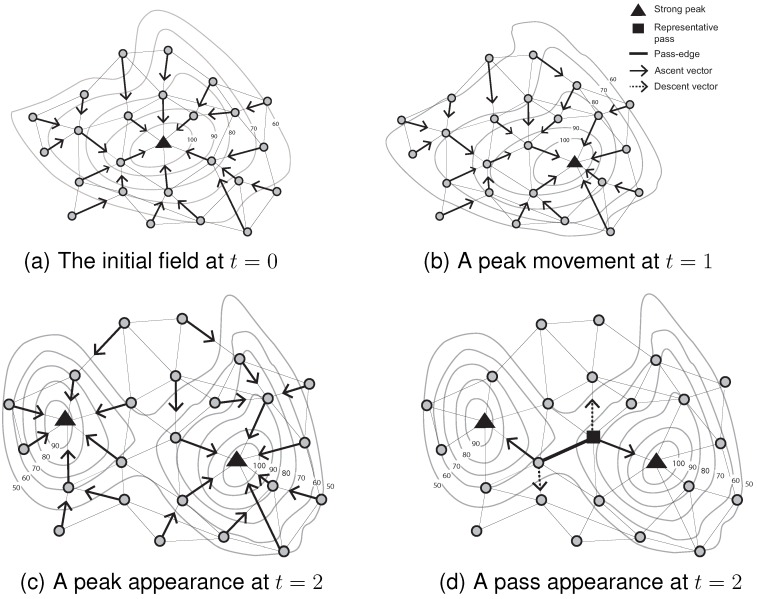
Monitoring events occurring on critical points between two consecutive time steps.

Based on Algorithms 1–4, Figure 4 summarizes the mechanisms for initializing and monitoring peak movement and appearance events (with associated pass appearance) over three consecutive time steps. Figure 4a shows the initial identification of a peak in the field. The ascent vectors of all of the nodes flow into the single peak. Next, as the scalar field evolves at $t_{1}$, a different node becomes a peak. Its peak identifier, however, remains unchanged (see Algorithm 2). A dramatic change of the scalar field leads to the appearance of a peak in Figure 4c. The previous catchment area at $t_{1}$ is divided into two catchment areas at $t_{2}$. The ascent vectors of all of the nodes partition the network into two groups (see Algorithm 4). The appearance of a new peak also entails the appearance of a new pass in Figure 4d. The appearance (disappearance) of passes is dependent on the appearance and disappearance of peaks/pits [10]. Passes cannot appear independently of peaks and pits. This fact forms the basis of our pass monitoring mechanism, explained in the following section.

### 4.2. Monitoring Events Occurring on Passes

Peak (pit) appearance and disappearance events lead to the detection of pass appearance and disappearance events. As illustrated in Figure 4, the appearance and disappearance of a pass is entirely dependent on the appearance and disappearance of peaks or pits. When a peak disappears (Algorithm 3, Line 16) or appears (Algorithm 4, Line 39), the affected node will update its peak identifier and inform its neighbors of the change. Each node can also update its pit identifier using a similar pattern for pit events. By broadcasting those events, using for example swpk messages, nodes can recognize whether they are involved in pass-edges. For example, assume one of two peaks connected by a pass disappears between consecutive time steps. If there is a representative pass between two peaks, this node is no longer a pass because one of the associated peaks has disappeared. A representative pass cannot preserve the pass-edges property (*i.e.*, two different peaks and two different pits). Thus, it is possible to infer a pass disappearance event after receiving a swpk message.

**Figure 5:**
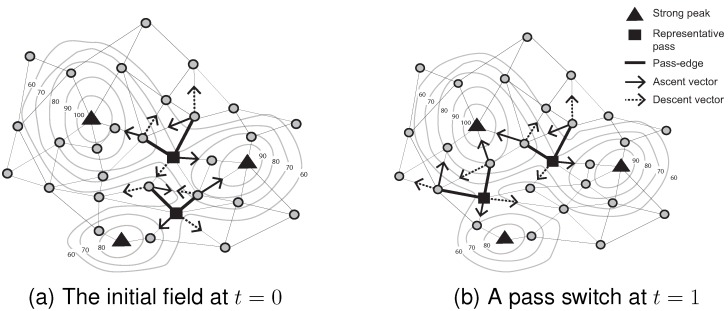
Switch event.

By contrast, pass movement and switch (the special topological event that occurs when the identities change of the two peaks and two pits associated with a pass) may occur without any events occurring on the pass’ associated peaks or pits. For example, in Figure 5, a pass moves and switches between two consecutive time steps, even though no events occur on the associated peaks (*i.e.*, no peak appearance, movement or disappearance).

Algorithm 5 highlights the main features of pass switch and movement monitoring. When a pass-edge node receives notification of a sensed-value or peak-identifier change from a neighbor (Algorithm 1, Line 19), this system event triggers a refresh of the pass-cluster and representative pass. If there is a change of members in a pass-cluster in addition to a change in the representative pass, the pass-edge node broadcasts a uppc message to its neighbors. This message reconciles the pass-cluster and representative pass of neighbors that are all associated with the same peaks and pits. For example, when a representative pass node becomes a regular node, it sends a wirp message to monitor events occurring on new passes (Algorithm 5, Line 7). If a new representative node receives a wirp message, it can infer what event occurred on its associated pass, such as movement or switch (Algorithm 5, Line 12). If the associated peaks and pits are different, a switch event has occurred. Conversely, if the associated peaks and pits are the same, a pass movement event is confirmed. **Algorithm 5** Monitoring events occurring on passes.1: Fragment extend: Algorithm 1, 2, 3, 42: Local variables: list of passes, Passes, initialized empty; list of pass cluster, *Passcluster*, initialized empty; list of a representative pass, *Repass*, initialized empty; list of a last representative pass, ${\mathrm{Repasse}}_{l}$, initialized empty;3: *Receiving* (uppc, *i*, Npasscluster)4:  **if**
Passes have the same associated peaks (pits) **then**5:   **update**Passcluster and Repass6:   **if**
Repass=∅ and ${\mathrm{Repasse}}_{l}\neq \emptyset$**then**7:    **send** (wirp, $\overset{˚}{\mathrm{id}}$, Passcluster) to a node *i*8:   **if**
Passcluster≠Npasscluster
**then**9:    **broadcast** (uppc, $\overset{˚}{\mathrm{id}}$, Passcluster)10: *Receiving* (wirp, *i*, Nrepass)- -
*Infer pass events*11:  **if** this node is a representative pass **then**12:   infer events occurring on a pass13:  **else**14:   deliver a wirp message to a representative pass.

### 4.3. Scalability

During the ongoing monitoring, sensed-value changes at a node trigger an upd8 message to neighbors. This message may in turn trigger a finite number of further messages for monitoring events occurring on the surface network (*i.e.*, udsf, wipk, cetc, etcl, cebc, wnpk, ispk, rspk, swpk, uppc or wirp). However, as surface events are expected to be relatively rare, in comparison to changes in the state of the field, such messages are expected to have a much smaller effect on scalability. In other words, the worst case is that all surface events occur simultaneously. In reality, it is rare that this happens. All messages for monitoring events therefore are not necessary at each time step. Overall, it is to be expected that between any two time steps, the algorithm will generate approximately |V|+k messages, where *k* is the number of messages for monitoring events occurring on surface networks. The exact number *k* will depend strongly on the specific details of the types of changes occurring, although given that the sparsity of events is expected to be much smaller than |V|. As a result, the overall communication complexity of the algorithm is expected to be linear in the number of nodes, O(n). However, due to the dependence on the events that occur, this expectation must be tested experimentally, as in the following section.

## 5. Experiments

The algorithm described in the previous section was evaluated with respect to four key features (overall scalability, latency, load balancing and accuracy). In terms of accuracy, this paper compared the proposed algorithm’s results with the results obtained from two centralized algorithms for surface network derivation (*i.e.*,^29^,^31^]).

### 5.1. Experimental Setup

The algorithm for monitoring spatial events occurring on surface networks was implemented within the agent-based simulation system, NetLogo [32]. A randomized scalar field was generated and evolved continuously in the NetLogo system. The randomized field was constructed from kernel density smoothing applied to randomly-moving particles. The approach allowed the generation of evolving randomized surfaces across a range of surface roughness levels.

By varying the kernel density smoothing parameters, surfaces with varying degrees of surface roughness were generated. For the ease of comparison, the surface roughness was classified at four levels. Level 1 surfaces had, on average, 6 critical points; Level 2, on average, 8 critical points; Level 3, on average, 14 critical points; Level 4, on average, 26 critical points. There are no special thresholds to differentiate the surface roughness. This classification is based on the computational complexity.

Each generated surface was allowed to evolve for ten simulation time steps, inclusive of the initial step. Geosensor networks were also simulated at five sizes, ranging from 1000–16,000 nodes. The network was connected by a unit disk graph (UDG), and node locations in the network were randomly distributed. The level of network connectivity (*i.e.*, average node degree) was kept constant to ensure comparability across the different network sizes.

The total number of simulations therefore was 4 surface levels × 5 network sizes × 10 replications × 10 (surface evolution scenarios) = 2000. The performance of the algorithm was documented for each simulation scenario.

### 5.2. Overall Scalability

**Figure 6:**
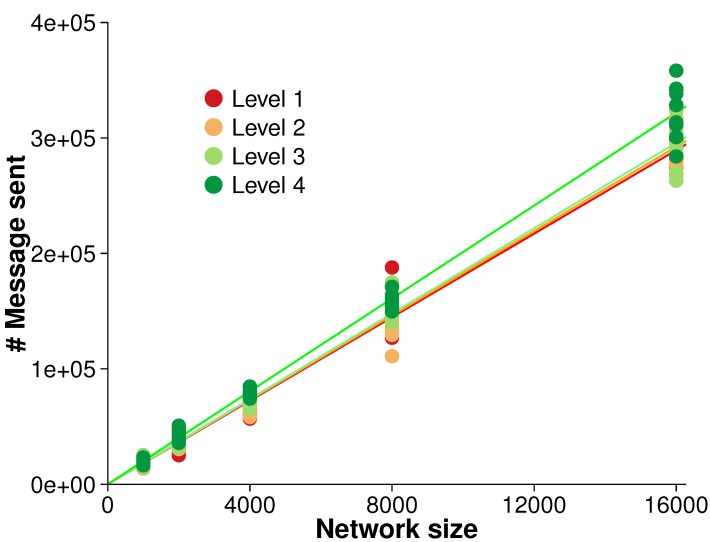
Overall scalability for monitoring events (averaged over 10 randomized networks and 10 consecutive time steps in each evolution).

The efficiency of the algorithm was evaluated with regards to overall scalability. There were five different network sizes (*i.e.*, 1000, 2000, 4000, 8000 and 16,000 nodes). As the network size increased, the number of messages sent was measured. A strong linear relationship between the network size and the number of messages sent was observed, as shown in Figure 6. A linear regression over the different surface roughness levels indicated that each node generated between 18.09 and 20.12 messages over the simulation, $R^{2}\geq 0.99$ in all cases. The close fit to a linear regression is in accordance with our expectation of overall O(n) scalability (see Section 4.3).

**Figure 7:**
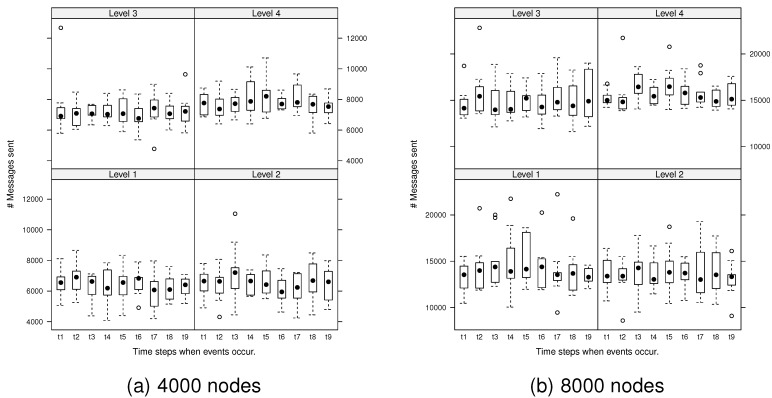
Number of messages generated by spatial events at each evolution step: dots are deemed to be outliers.

Next, we explored the variability in the number of messages generated by the algorithm during ongoing changes. At each evolution time step, the number of messages required to monitor any events that occurred was investigated. Figure 7 presents the number of messages generated by spatial events at each evolution time step for the network, 4000 and 8000 nodes.

A mixed-factorial ANOVA test was used to evaluate the significance of any differences in messages generated due to surface roughness levels, evolution time steps and interaction of surface roughness levels and evolution time steps. Accordingly, there are three null hypothesis:

H1 There is no main effect of surface levels considered separately.

H2 There is no main effect of evolution time steps considered separately.

H3 There is no interaction of surface levels and evolution time steps considered together.

The ANOVA test revealed that there were significant differences in the messages generated between the different surface levels at the 95% confidence level (*i.e.*, rejection of H1). For example, F(3,36)=12.41, p<0.05, $\eta_{G}^{2}=0.26$ for 4000 nodes and F(3,36)=4.24, p=0.011, $\eta_{G}^{2}=0.14$ for 8000 nodes. $\eta_{G}^{2}$ (generalized eta-squared measure) indicates the effect size: the practical degree of difference between groups [33]. For 4000 nodes, the effect size is large, and for 8000 nodes, the effect size is medium. Thus, the effect between surface levels is meaningful, both statistically and practically.

The *post hoc* comparisons (using the Tukey honestly significant difference [34]) indicated that there were no significant differences between Level 1 and 2 surfaces (p=0.198) or between Level 3 and 4 surfaces (p=0.106) for 8000 nodes and Level 1 and 2 surfaces (p=0.717) for 4000 nodes. However, there were significant differences between all other pairs of surface levels (p<0.05). There was however no significant difference between each evolution time step (no evidence to reject H2) and no significant interaction between surface levels and evolution time steps (no evidence to reject H3). Other network sizes exhibited the same trends (*i.e.*, significant differences in messages generated between surface roughness levels and no significant differences between evolution time steps or interactions between surface levels and evolution time steps).

### 5.3. Latency

The operational latency of our algorithm is the time delay between an event occurring and that event being detected. Even an efficient algorithm may suffer from long latencies. Understanding operational latency can give a picture of an algorithm’s practical usability and associated efficiency tradeoffs.

**Figure 8:**
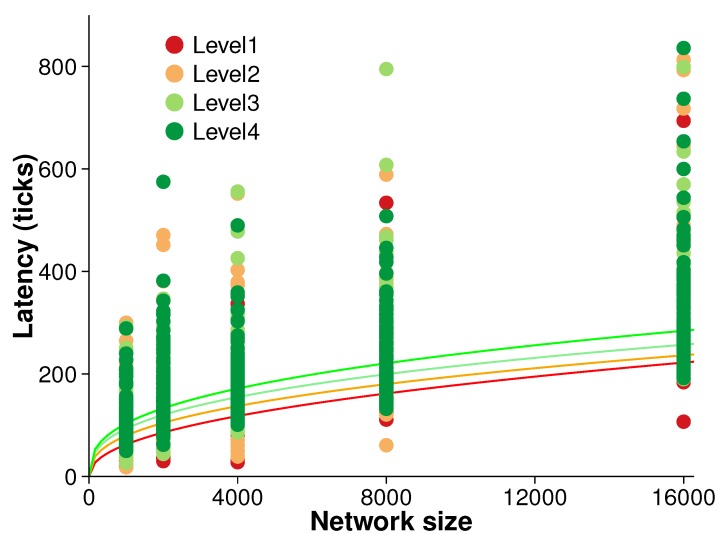
Latency (averaged over 10 randomized networks and 10 consecutive evolution).

Figure 8 presents the results of an experiment measuring the latency of the algorithm. Latency was measured during each evolution time step over the five different network sizes and four different surface roughness levels. Ten randomized replications were conducted at each surface level.

There is broadly a trend of increasing latency with both network size and surface roughness. However, that trend is not clear. A simple power regression reveals only moderate correlation between latency and network size (*i.e.*, $0.448\leq R^{2}\leq 0.559$). Further, non-parametric tests confirmed the initial inference that latency is not strongly associated with the network size.

**Figure 9:**
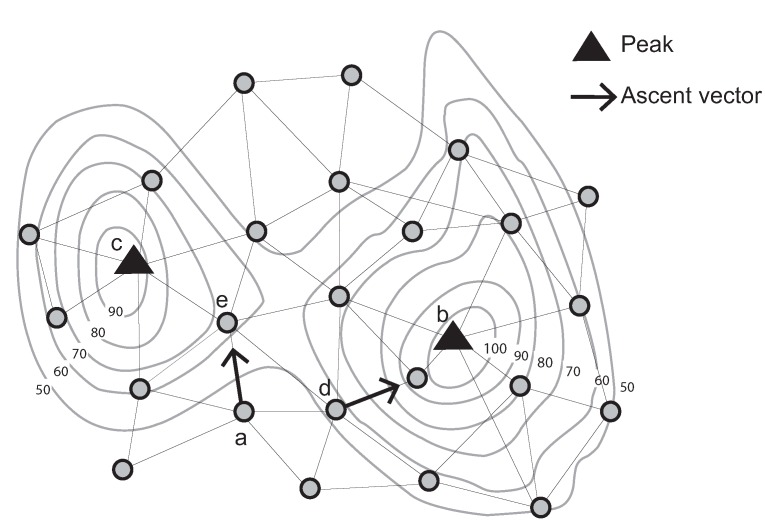
Example asynchronous update problem. The node *a* was previously a peak.

In fact, latency is more closely related to the types of events resulting from network asynchronicity. As this paper makes no assumptions about message ordering nor about communication delays, messages are assumed to be reliably delivered in a finite amount of time. Such minimal assumptions help to increase the robustness of decentralized algorithms. However, when decentralized spatial algorithms monitor events in a dynamic field, asynchronous algorithms have difficulty in coordinating nodes in terms of data consistency and efficiency. For example, Figure 9 illustrates one such problem of network asynchronicity. Node a is a peak at time $t_{1}$. At time $t_{2}$, this peak moves to node c. In order to monitor this peak movement, node a should send a wipk message via its ascent vector. Unfortunately, due to asynchronous communication, a peak movement event can be misidentified as a peak disappearance and a new peak appearance event. For example, assume at time $t_{2}$ the ascent vector of node a points to *d* as soon as receiving information from node d. In that case, a may send the message to node d before receiving all of the necessary information from its neighbors to correctly update its ascent vector (to point to e). In that case, node a will detect a peak disappearance event and node c will detect a new peak appearance. These misidentified events cause the replacement of peak identifiers and can result in longer latency.

### 5.4. Load Balance

Load balance is a vital factor in network longevity. Resource-constrained geosensor networks are vulnerable to uneven load balance, causing holes in network coverage.

As already mentioned, initialization requires a ping handshake message and at least two further messages, requiring 3|V|+m messages, where *m* messages are required for identifying the critical points, m≪|V|. During ongoing monitoring, each node must send at least one message for updating its sensed value and additional messages for monitoring events, requiring |V|+k at each evolution, where k≪|V|. In this study, each generated surface evolved for ten simulation time steps (*i.e.*, initialization and nine ongoing monitoring). Thus, each node is expected to require approximately 12|V|+m+9k over our entire simulation. It is expected that uneven load balance is more likely to be associated with rougher surfaces.

**Figure 10:**
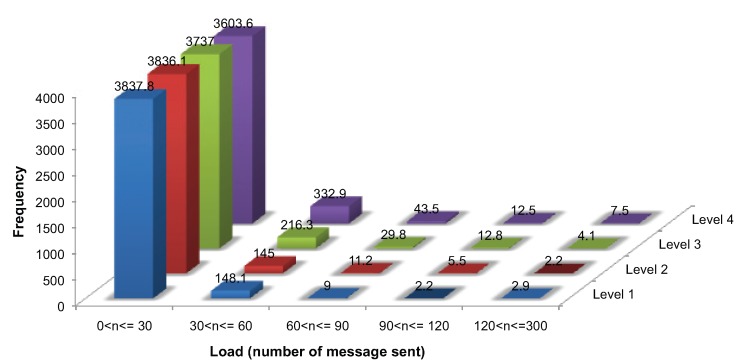
Load balance for communication messages (averaged over 10 networks of 4000 nodes and 10 consecutive evolution).

Figure 10 presents the load balance for 4000 nodes. While a considerable number of nodes sent fewer than 30 messages, a few nodes transmitted more than 250 messages, 267 in the worst case. One-way ANOVA was used to analyze the difference in the proportion of nodes with a load of less than 30 messages between the four surface levels. The test revealed that there was a difference, significant at the 95% level (F(3,36)=11.83, p<0.05, $\eta_{G}^{2}=0.49$), between the roughest (Level 4) surface and the other surfaces (Levels 1–3). However, no significant differences were found among Level 1, 2 and 3 surfaces. Thus, there is evidence that the roughest surfaces lead to significantly different load balances. However, the algorithm is relatively tolerant to moderate changes in surface roughness levels, which lead to no significant change in load balance. The same results were obtained for all of the different network sizes.

### 5.5. Accuracy

An efficient algorithm is only useful if it can accurately identify the events occurring. The accuracy of the algorithm was measured using standard information retrieval measures: positive predictive value (PPV, also called precision), recall and F1-score [35,36].

To provide a comparison in assessing accuracy, two standard centralized algorithms [29,31] were combined to generate the ground truth for each simulated surface. Each algorithm has its advantages and disadvantages. The algorithm of [29] can identify critical points using the logical simple comparison of neighbors. It is, however, well known that this local approach is sensitive to minor, small-scale variations in the surface (see [31]). Such minor variations can produce spurious critical points. In order to minimize spurious critical points, [31] models a surface using a quadratic equation. Morphometric parameters derived from the quadratic coefficients, such as slope or maximum and minimum convexity, can then be used to identify critical points. This method uses variable kernel size (window size) to identify critical points. Even though this feature has a positive effect on the identification of critical points at various scales, there are no guidelines on the ideal size of the kernels for the identification of critical points. Thus, the critical points identified by the two approaches can be inconsistent. Therefore, in this paper, the algorithm of [29] was firstly used to identify critical points, and the morphometric characterization [31] was combined to exclude spurious critical points for the best possible classification of surface networks.

Further, edge effects tended to lead to poor results for all algorithms at the boundary of the network (critical points can be falsely identified due to discontinuity at the boundary of the geosensor network). Consequently, nodes within one-hop average communication distance of those boundaries were connected to a “virtual” pit to remove edge effects. This approach was applied consistently to all different network sizes and across all tests.

In terms of the F1-score for the identification of critical points, the F1-score increases as the network size increases. The detection of peaks and pits tends to perform better than passes. This is expected, as the identification of passes is based on that of associated peaks and pits. Thus, pass identification is more difficult in a decentralized algorithm.

It should be noted, however, that based on the F1-score, the algorithm occasionally performed better at monitoring events on passes than on peaks and pits, as shown in Table 1. This is because the F1-score calculated for the identification of passes already reflected the effect of the identification of peaks (pits). When the F1-score for monitoring events occurring on passes was computed, passes identified by the algorithm were only considered. In terms of the F1-score for monitoring events on passes, unidentified passes were not included in order to avoid double-counting errors.

**Table 1:** F1-score for monitoring events occurring on peaks and passes on a Level 4 surface

Network sizes	1000	2000	4000	8000	16,000
Peak	0.77	0.86	0.89	0.93	0.94
Pass	0.84	0.91	0.90	0.88	0.91

**Figure 11:**
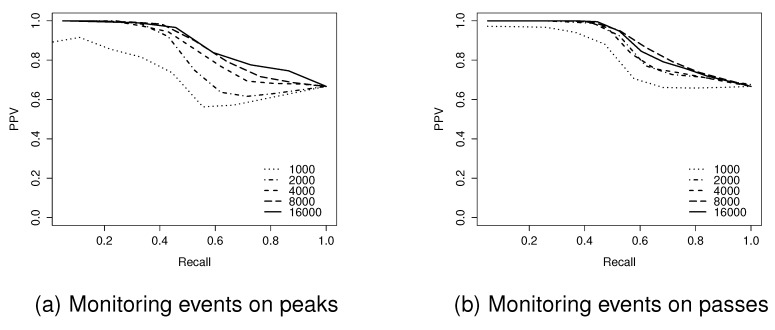
Positive predictive value (PPV)-recall curves for network sizes of 1000–16,000 nodes on a Level 4 surface.

The F1-score provides a global view of the performance of the algorithm. However, it cannot provide further details of the inverse relationship between PPV and recall. Figure 11 examines in more detail the trade-off between PPV and recall, using PPV-Recall curves for the Level 4 surface, ranging from 1000–16,000 node network sizes. These results indicate that at the highest levels of recall, all of the events achieve similar, relatively high levels of PPV (typically >0.6). By contrast, the highest levels of PPV are dependent on network size, with larger networks being capable of reaching higher levels of PPV. This implies that due to the networks’ coarse granularity, the algorithm cannot guarantee that all true events are detected, although most detected events are correct.

Hypothesis testing confirmed that this apparent effect was statistically significant. The null hypothesis, that there is no effect of network size upon the F1-score for monitoring events at peaks on the level 4 surfaces, was rejected (Kruskal-Wallis test, p<2.2e-16 significant at the 95% level). More dense network sizes achieved better performance in terms of monitoring events occurring on peaks. This pattern of increased performance was repeated for all surface levels.

## 6. Discussion

The results of the experiments on overall scalability confirmed that the algorithm is in practice scalable, with overall O(n) communication complexity. The experimental results indicated a good fit, with $R^{2}\geq 0.99$ in all cases. The results also indicate a significant effect of surface roughness, as might be expected, with rougher surfaces leading to greater numbers of messages generated and, to a lesser extent, to greater latency. We can conclude that the algorithm can always be relied on to perform efficiently, even though efficiency is expected to decrease for increasingly rough monitored surfaces.

The latency of the algorithm appears primarily to be related to the type of events occurring. For example, if a peak disappears, nodes that have an out-of-date peak identifier must exchange their peak identifier for the new one. This swapping can lead to a long latency compared to movement or switch events of existing critical points and edges. The problem is exacerbated by the assumption of an asynchronous network (Section 5.3). An efficient synchronous network (e.g., with bounded communication delays) is beneficial for data consistency and efficiency [37,38] and, in our case, could help improve latency.

The third evaluation criterion was load balance. While a substantial number of nodes transmit fewer than 30 messages over the entire simulation (10 time steps), a small number of nodes sent more than 200 messages in that period. These high-load nodes were frequently located at a pass-cluster. Nodes at the pass-cluster may sometimes need to repeatedly update their pass-cluster members and their representative (or strong) pass. Future work should improve the algorithm by looking specifically at improved mechanisms to reduce the updates required at pass-clusters.

The accuracy of the identification of critical points is high, comparable to that of centralized algorithms. The results demonstrate that an increased network size is associated with improved accuracy, as might be expected due to increased sensor detail about the surface. In the case of larger networks, the F1-score for event detection reached over 0.95; but even in the worst cases of the smallest networks, F1-scores remained about 0.6. In terms of key future work, it is interesting to investigate how the algorithm behaves toward the presence of noise. Our related work [39] already investigated the robustness of the algorithm to the inaccuracy of sensors in a static field. However, the robustness of the algorithm should be further investigated for a dynamic field.

## 7. Conclusions

This paper has presented a decentralized algorithm that can efficiently and accurately identify events occurring on a surface network. The surface network is derived from a scalar field, monitored by a geosensor network without requiring any coordinate localization. Our approach to event detection is to first focus on the changes occurring in catchment areas. Changes to these areas are the basis for monitoring the appearance, disappearance and movement of peaks and pits. In turn, the appearance and disappearance events at peaks and pits are used as the basis for the detection of appearance and disappearance events at passes. Finally, the fourth event type, switch (where the edges of the surface network change even though no changes may occur on the associated peaks and pits) are captured by monitoring each node’s ascent/descent vectors.

The approach presented in this paper has wide applications to environmental monitoring. Even though this paper focuses on empirical evaluations, the algorithm proposed might be implemented and tested in a real sensor network as a result of advanced, more economical technology, such as thousands or millions of nodes. For example, a wildfire risk management system might be required to monitor the highest risk areas, not just simply through arbitrary thresholds, but by tracking the appearance, disappearance and movement of high temperature hotspots or the local fuel moisture minimum. These changes can be efficiently and intuitively summarized as qualitative events occurring on surface networks, with, in turn, important events and changes reported back to wildfire monitoring decision makers and systems.
